# Knowledge and attitude on family planning among medical students in Egypt: a multicentric cross-sectional study

**DOI:** 10.1186/s12889-024-20827-9

**Published:** 2025-05-28

**Authors:** Hajer Azzam, Basma Kamel, Ahmed Esawy, Mariam Awadh, Toka Rabea, Nouran Riad, Abdullah Suliman, Tasneem Deibes, Eman Ayman, Aly Elbaz, Farah Ashraf, Doaa Alemam, Data Contributors, Ahmed Mansour, Ahmed Mostafa Amin, Mohamed I. Mohamed, Rodina N. ElWakil, Youssef Elhdad, Mohamed Mohalal, Rana Ashraf, Sohila Elshabrawy, Mahmoud Elkaffas, Enjy M El-bialy, Nora I Yousef, Kareem M Arafa, Osama A. Abd Elaziz, Yasmeen Doma, Lara M. Saeed, Rawan E. Elnaggar

**Affiliations:** 1 Integrated Program, Faculty of Medicine, Mansoura University , Mansoura, Dakahlia Governorate Egypt; 2 Mansoura Manchester Program for Medical Education, Faculty of Medicine, Mansoura University, Mansoura, Dakahlia Governorate Egypt; 3 Mansoura Students Scientific Association (MSSA), International Federation On Medical Students Association -Egypt (IFMSA-Egypt), Faculty of Medicine, Mansoura University, Mansoura, Dakahlia Governorate Egypt; 4 Public Health and Community Department, Faculty of Medicine, Mansoura University, Mansoura, Dakahlia Governorate Egypt

**Keywords:** Attitude, Contraception, Egypt, Family Planning, Knowledge, Medical students

## Abstract

**Background:**

Family planning refers to a conscious effort done by a couple to limit or space the number of children they have through the use of contraceptives. Effective family planning can prevent abortions, maternal morbidity, and maternal deaths. It contributes to broader public health goals, including improved maternal and child health outcomes. Studies have shown that medical students need more knowledge regarding family planning. Enhancing their understanding of family planning can positively influence their future clinical practice and patient counseling, leading to better healthcare delivery in their communities. Thus, this study aimed to assess the knowledge and attitude of medical students in Egypt towards family planning and the factors affecting them.

**Methods:**

This was a multi-centric descriptive cross-sectional study with an analytical component. We used a multistage stratified cluster sampling method. Twelve Egyptian medical universities were chosen to ensure the representation of governmental, private and Al-Azhar medical schools. The estimated sample size was 1072. Our tool was a self-administered questionnaire used from a previously published study, which showed acceptable reliability: α = 0.825 for knowledge and 0.906 for attitude. We analyzed data using IBM Statistical Package for Social Science (SPSS) software version 25. Results were statistically significant if the *p*-value was ≤ 0.05.

**Results:**

We surveyed 926 medical students, aged 17 to 27 years (mean 21.42 ± 1.90 years), with a balanced gender distribution (52.15% males). The majority showed poor knowledge about family planning (85.9%), and inappropriate attitudes (52.7%). We also found that gender significantly influenced knowledge levels (*p* = 0.04), with higher female scores. While the academic year significantly affected attitudes (*p* = 0.05). Female sex was a significant predictor of good knowledge (COR = 1.47, 95% CI = 1.01–2.14, *p* = 0.05).

**Conclusion:**

Our overall scores of knowledge and attitude towards family planning were low. Female gender was a significant predictor of good knowledge. Academic year significantly affected the attitude with higher appropriate attitudes among 4th-year students. Stakeholders concerned with family planning should work together to bring behavioral changes towards family planning by providing information, education and communication.

**Supplementary Information:**

The online version contains supplementary material available at 10.1186/s12889-024-20827-9.

## Background

Family planning is a critical aspect of reproductive health that allows couples to determine the number and spacing of their children via contraception [1]. Contraception denotes the use of various methods to prevent pregnancy during sexual activity, including options like birth control pills, condoms, and surgical procedures [2]. The American College of Obstetricians and Gynecologists (ACOG) underscores the role of family planning in managing pregnancy timing and intervals, marking its effectiveness in averting many negative health consequences [3, 4].

Effective family planning can prevent up to 32% of maternal deaths, 90% of abortions, and 20% of maternal morbidity globally [5]. Moreover, family planning encourages healthy practices, such as the intake of folic acid prior to conception, which has been shown to reduce the incidence of fetal neural tube defects by up to 75% [2]. In addition to the health benefits of family planning, it also has multiple economic and social advantages. It slows population growth and improves income potential, reducing poverty and improving quality of life [6]. The World Health Organization (WHO) recommends three-year birth intervals to help optimise maternal and child health outcomes [7].

Contrarily, closely spaced pregnancies are associated with increased risks of complications such as eclampsia, systemic infections, low birth weight, and preterm delivery. Women who experience such complications are at a higher risk of discontinuing education, earning less, and living in poorer households [8].

Despite family planning advantages, many women around the world lack accurate information about contraceptive options. An Indian study showed that the majority of women in their child-bearing age either know very little or have false information about contraception [9]. Even when they are aware of certain contraceptive methods, many are unsure of how to use them correctly or where to obtain them [9]. Similarly in Lebanon and Indonesia, declines in contraception use highlight the urgent need for the promotion of family planning to support health and development in their respective communities [10, 11].

University students are at the age where many may become parents, so their knowledge regarding this topic is vital [12]. They are also faced with many interests regarding their career paths and marriage, especially medical students who will be the future doctors responsible for giving reliable counselling to patients [13, 14]. Studies in many countries assessed the knowledge and attitude of medical students. In Nicosia and Northern Cyprus, an evaluation of an international medical school revealed that the knowledge of first-year medical students on family planning was insufficient [15]. Another study in Romania showed that medical students believed it was important to be informed on this subject [16]. Moreover, a recent Italian study revealed that female medical students needed sexual and contraceptive counselling despite their scientific knowledge [17].

Many factors could affect students’ attitudes towards family planning, such as gender and religion [18, 19]. Whether the students come from urban or rural areas, or being in a private or a public college has a great impact too [14]. In addition, cultural and social barriers could thwart contraception use in women [20]. A recent study in Ethiopia points to several reasons why family planning isn’t used, including provider bias, lack of access to contraceptive methods, poor understanding, fear of adverse effects, and acceptance based on social and religious views [21].

Accordingly, interventions such as education and training for current and future healthcare providers should be investigated to address these problems. Previous training for health care providers in Nigeria successfully improved the quantity and quality of family planning services provided at private healthcare facilities [22]. In addition, many studies have shown the impact of family planning education on medical students. Studies conducted in Malaysia and India indicated that medical students who initially had inadequate training regarding family planning, had their knowledge significantly improved following targeted educational interventions [13, 14]. Another study conducted in Chicago on third-year medical students emphasised how necessary it is to develop high-impact curricula for teaching family planning to medical students and discussed how team-based learning is an innovative teaching style as a learning strategy for family planning [23].

Egypt has its own contributions to this issue. The rapid population growth in Egypt since the 2000s has affected maternal and child health outcomes, education and economy particularly due to limited access to family planning services [24]. In response, both government and non-governmental organizations are actively involved in promoting the importance of family planning within society through various initiatives. The Ministry of Health has launched The National Population and Development Strategy (2023–2030) to raise awareness of family planning, aligning with Egypt’s 2030 Sustainable Development Vision and the National Project for the Development of the Egyptian Family (NPDEF) [25].

Additionally, non-governmental organisations such as the International Federation of Medical Students’ Association - Egypt (IFMSA-Egypt) play a role as stakeholders in family planning. It empowers medical students to advocate for family planning through community awareness activities that enhance public well-being [26]. IFMSA-Egypt has established local committees in medical faculties throughout Egypt to address these shared goals to capacitate medical students for addressing such topics in the community [27].

### Study rationale

Despite these efforts, there has been no prior research evaluating the knowledge and attitudes toward family planning among Egyptian medical students.

Therefore, we aim to address the critical gap in the literature concerning this matter and evaluate the knowledge of Egyptian medical students about family planning and their attitudes exploring the factors that may affect them.

Our study will provide the concerned stakeholders like IFMSA-Egypt with evidence they can use to plan their advocacy and capacity-building activities regarding their main target population, medical students in Egypt. In addition, family planning initiatives in Egypt will benefit from our findings by highlighting the defect in the perception of the future applicators and providers of family planning practices. This will subsequently help to develop targeted educational programs, improve the medical curricula, conduct awareness campaigns and workshops, and create policies to call for action that will enhance reproductive health outcomes and the overall quality of life.

## Methods

### Study design, aim and setting

A multicentric, descriptive cross-sectional study with an analytic component was conducted among undergraduate medical students from October 2023 to January 2024 at 12 different Egyptian universities to assess their knowledge and attitude towards family planning.

### Study sampling and sample size

A multistage stratified cluster sampling method was used to collect a randomized sample from the universities we included. First, we divided Egypt into five geographical regions (Delta, Capital, East, West, and Upper Egypt). To ensure a good representation of our sample to the Egyptian population, we randomly chose two or three universities from each region. Those randomly chosen 12 universities were further categorized according to their type into nine governmental ones and three from the private and AL-Azhar sectors.

The governmental faculties of medicine included Mansoura, Tanta, ElMenofia, Aswan, Port Said, Alexandria, Helwan, and Suez Canal. The private one was Misr University (MUST) and El Azhar Cairo boys and girls were included.

Subsequently, we obtained the name lists of all medical students enrolled in the chosen faculties from the first till the intern academic years. An additional randomization was applied to select the included clusters of students represented by their subgroups in each level.

We calculated our sample size online using Open-Epi calculator by assuming a 50% frequency (p), a 5% margin of error, and a 95% confidence interval [28]. The minimum required sample size was 383 which was then multiplied by 2 to compensate for the cluster sampling technique’s design effect, and a 40% drop rate was added to compensate for potential dropouts and incomplete responses. Thus, the final sample size was 1072. Complete 926 responses were collected with an 86℅ response rate.

### Data collection and study questionnaire

Our tool was an English-language, self-administered reliable questionnaire from a previously published study assessing the knowledge and attitude towards family planning among Yasoujian women in Yasouj City, Iran [29]. Both knowledge and attitude were assessed in our study among medical students in Egypt.

Before we distributed the questionnaire, a public health professional reviewed it to ensure its content and validity. We then conducted a pilot study involving 30 students to validate the questionnaire’s reliability and ensure its clarity. We assessed the reliability of the knowledge and attitude scales using Cronbach’s alpha test, which measures the internal consistency of the items. A Cronbach’s alpha value greater than 0.7 indicates that the scale demonstrates acceptable reliability for the intended use. The knowledge scale consisted of 23 items with an acceptable value (α = 0.825). The attitude scale consisted of 15 items with an acceptable value (α = 0.906). The data from the pilot study were excluded from the final analysis.

 The questionnaire was conducted via online Google Forms and consisted of four sections: consent, sociodemographic data, knowledge of Family Planning, and attitude toward Family Planning. Section 1 included informed consent where participants were asked to indicate whether they agreed or disagreed to participate in the survey. Section 2 collected information about the participants’ sociodemographic characteristics. Participants were asked to provide their age, sex, academic year, university, academic grade range in the past years, residency (urban or rural), living condition, nationality, religion, whether they work besides studying due to income needs, marital status, and the number of children they have if married. Section 3 assessed the participants’ knowledge about family planning. Participants were presented with a series of statements related to family planning. They were asked to indicate whether they believed the statement was true, or false, or if they were unsure. The statements covered various aspects of family planning methods, their effectiveness, side effects, and related misconceptions. Section 4 explored the participants’ attitudes towards family planning. Participants were presented with a series of statements related to family planning, and they were asked to indicate their level of agreement or disagreement with each statement on a Likert scale ranging from “Strongly Disagree” to “Strongly Agree.” The statements covered topics such as the importance of family planning, shared responsibility, health benefits, and perceptions about specific family planning methods. The questionnaire was designed to be completed within approximately 5 min.

Two collaborators were selected from each included university. Collaborators distributed the questionnaire online to the students chosen through their contacts, providing a thorough explanation in advance to ensure their understanding.

### Ethical approval

Approval was obtained from the IRB Committee of the Faculty of Medicine, Mansoura University (R.22.091867). The study was executed by the ethical standards set in the 1964 Declaration of Helsinki and its later modifications. Informed consent was obtained from all the subjects involved in the study. The survey cover page had an "agree" option, where eligible students who accessed the electronic survey should click on it before completing the survey questions. We ensured anonymity and confidentiality for participants.

### Statistical analysis

The data collected from the questionnaire were analyzed using IBM Statistical Package for Social Science (SPSS) software version 25. Knowledge and attitude scores were calculated and categorized into good or poor for knowledge and appropriate or inappropriate for attitude according to the median with a 75℅ cut-off value. Categorical data were summarized and described as numbers and percentages. Continuous data were summarized and described as mean and SD according to normality plots. Categorical and continuous data were compared using Chi-Square and independent t test, respectively. We used Pearson for the correlation between continuous variables. We calculated the crude odds ratio (COR) and their corresponding 95% confidence interval. Adjusted binary logistic regression was used to explore factors predicting good knowledge and appropriate attitude. Results were considered statistically significant if the *p*-value was ≤ 0.05.

## Results

Table 1 shows the sociodemographic characteristics of the 926 study participants. Their ages ranged from 17 to 27 with a mean of 21.42 ± 1.90 years. The number of male and female participants was almost equal with 483 (47.84%) females and 443 (52.15%) males. Students from all academic years took part in the study, with the majority 587 (63.4℅) in clinical years and the least 62 (6.7℅) being medical interns. Our sample consisted of students from governmental, private, and Al-Azhar universities. Most of them were in governmental universities (74.9%), while 12.6% were enrolled in private universities and 12.4% in Azhar universities. Almost half of the respondents (54.4%) performed well in their studies with grades of A or A-, while the rest scored B or lower. Most students lived in rural areas (63.6%), as well as the majority lived with their families (78.9%), and only 101 (10.9℅) worked besides studying for income needs. The participants were mostly Egyptian (94.4%), Muslim (96.9%), and single (94.7%).

### Primary outcomes

**Fig. 1:**
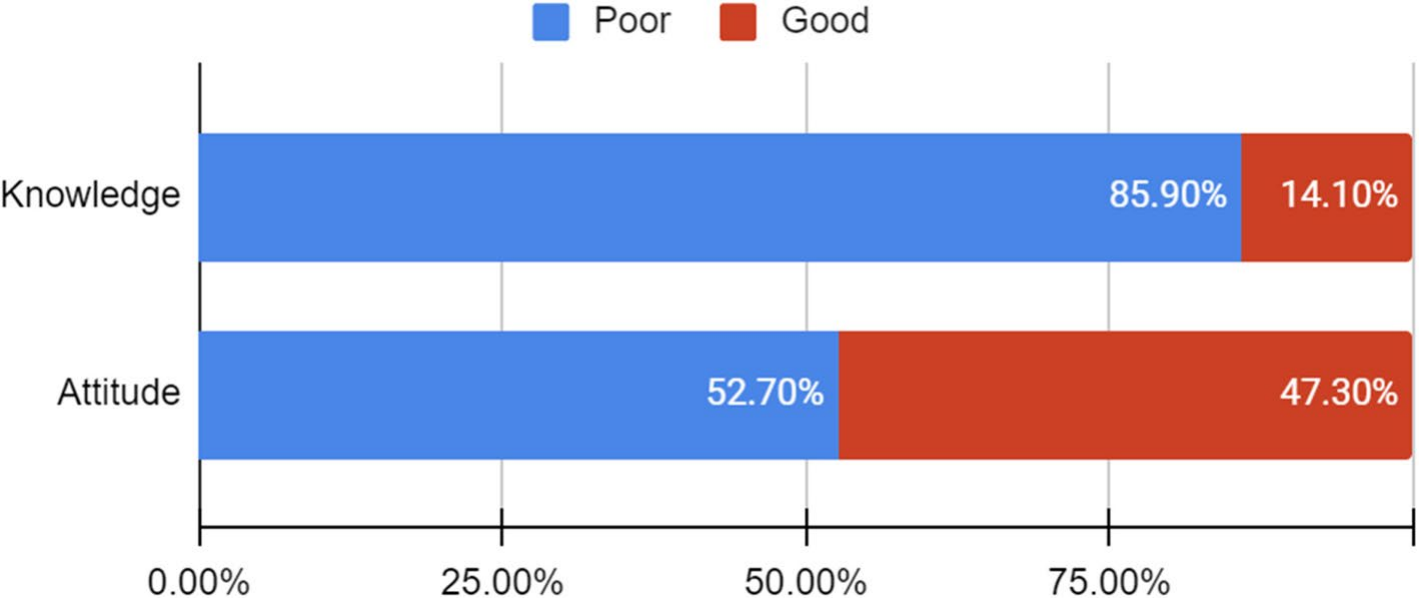
Prevalence of good and poor knowledge and attitudes among the studied group

This study aimed to assess the knowledge and attitude of medical students on family planning in Egypt. Our findings showed that the average knowledge score was 13.95 ± 3.7, with the majority of the participants having poor knowledge, 795 (85.9%) (Table 1; Fig. 1). The average attitude score was 53.78 ± 9.98 with almost equal numbers of appropriate and inappropriate attitudes, 438 (47.3%) and 488 (52.7%) respectively. Most of the participants reported that their source of information was the university curriculum,624 (67.2℅), followed by doctors, 413 (44.5℅), and social media, 302 (32.5%).

**Table 1:** Demographic characteristics, knowledge & attitude levels of participating students

	(*N* = 926)
Age (Years)	
Mean ± SD	21.42 ± 1.9
Min-Max	17–27
**Sex n(%)**	
Male	443 (47.84%)
Female	483 (52.15%)
**Academic year**	
**Basic Years n(%)**	277 (29.9℅)
1st year	128 (13.8℅)
2nd year	149 (16.1℅)
**Clinical Years n(%)**	587 (63.4℅)
3rd year	173 (18.7℅)
4th year	175 (18.9℅)
5th year	154 (16.6℅)
6th year	85 (9.2℅)
**Interns n(%)**	62 (6.7℅)
**University n(%)**	
**Governmental**	694 (74.9℅)
Alexandria	94 (10.2℅)
Aswan	103 (11.1℅)
El Menofia	76 (8.2℅)
Helwan	81 (8.7℅)
Mansoura	125 (13.5℅)
PortSaid	32 (3.5℅)
Suez	87 (9.4)
Tanta	96 (10.4)
**Private**	
MUST	117 (12.6℅)
**Azhar**	115 (12.4℅)
Azhar-Boys	65 (7℅)
Azhar-Girls	50 (5.4℅)
**Academic Grade n(%)**	
A, A-	504 (54.4℅)
B, B-	329 (35.5℅)
C, C-	80 (8.6℅)
D	13 (1.4℅)
**Residency n(%)**	
Rural	589 (63.6℅)
Urban	337 (36.4℅)
**Living Condition n(%)**	
Alone	72 (7.8℅)
With family	731 (78.9℅)
With friends/dorms	112 (12.1℅)
Other	11 (1.2℅)
**Nationality n(%)**	
Egyptian	874 (94.4℅)
Other	52 (5.6℅)
**Religion n(%)**	
Muslim	897 (96.9℅)
Christian	29 (3.1℅)
**Working beside studying because of income need n(%)**	
Yes	101 (10.9℅)
No	825 (89.1℅)
**Marital Status n(%)**	
Single	877 (94.7℅)
Engaged	45 (4.9℅)
Married	4 (0.4℅)
**Knowledge Score**	
Mean ± SD	13.95 ± 3.7
**Knowledge Class n(%)**	
Poor < = 75%	795 (85.9%)
Good > 75%	131 (14.1%)
**Attitude Score**	
Mean ± SD	53.78 ± 9.98
**Attitude Class n(%)**	
Inappropriate < = 75%	488 (52.7%)
Appropriate > 75%	438 (47.3%)
**Source of Information (multiple answers)**	
University Curriculum	624 (67.2℅)
Doctors	413 (44.5℅)
Social Media	302 (32.5%)
Relatives and friends	170 (18.3℅)
Awareness Campaigns	133 (14.3℅)
No source	89 (9.6℅)
TV	77 (8.3℅)
High school	18 (1.9℅)

### Factors affecting knowledge and attitude

The only factor having a significant effect on knowledge was gender with females having higher knowledge scores than males, 79 (16.4%), 52 (11.7%), *p* = 0.04 (Table 2). Other characteristics such as age, academic year, religion, and marital status did not significantly affect family planning knowledge among the participants.

**Table 2:** Factors affecting knowledge of family planning

	Poor Knowledge (*N* = 795)	Good Knowledge (*N* = 131)	*p*-value
**Age in Years mean (SD)**	21.43 (1.95)	21.31 (1.79)	0.5
**Gender n(%)**			
Male	391 (88.3%)	52 (11.7%)	0.04^a^
Female	404 (83.6%)	79 (16.4%)	
**Academic year**			
**Basic Years n(%)**	232 (83.8%)	45 (16.2%)	0.28
1st year	105 (82%)	23 (18%)	0.07
2nd year	127 (85.2%)	22 (14.8%)	
**Clinical Years n(%)**	512 (87.2%)	75 (12.8%)	
3rd year	157 (90.8%)	16 (9.2%)	
4th year	143 (81.7%)	32 (18.3%)	
5th year	133 (86.4%)	21 (13.6%)	
6th year	79 (92.9%)	6 (7.1%)	
**Interns n(%)**	51 (82.3%)	11 (17.7%)	
**University**			0.21
**Governmental**	603 (86.9%)	91 (13.1%)	
Alexandria	78 (83%)	16 (17%)	0.08
Aswan	89 (86.4%)	14 (13.6%)	
El Menofia	66 (86.8%)	10 (13.2%)	
Helwan	70 (86.4%)	11 (13.6%)	
Mansoura	116 (92.8%)	9 (7.2%)	
PortSaid	29 (90.6%)	3 (9.4%)	
Suez	75 (86.2%)	12 (13.8%)	
Tanta	80 (83.3%)	16 (16.7%)	
**Private**			
MUST	99 (84.6%)	18 (15.4%)	
**Azhar**	93 (80.9%)	22 (19.1℅)	
Azhar-Boys	58 (89.2%)	7 (10.8%)	
Azhar-Girls	35 (70%)	15 (30%)	
**Academic Grade n(%)**			
A, A-	435 (86.3%)	69 (13.7%)	0.98
B, B-	281 (85.4%)	48 (14.6%)	
C, C-	68 (85%)	12 (15%)	
D	11 (84.6%)	2 (15.4%)	
**Residency n(%)**			
Rural	512 (86.9%)	77 (13.1%)	0.22
Urban	283 (84%)	54 (16%)	
**Living Condition n(%)**			
Alone	61 (84.7%)	11 (15.3%)	0.53
With family	632 (86.5%)	99 (13.5%)	
With friends/dorms	94 (83.9%)	18 (16.1%)	
Other	8 (72.7%)	3 (27.3%)	
**Nationality n(%)**			
Egyptian	753 (86.2%)	121 (13.8%)	0.28
Other	42 (80.8%)	10 (19.2%)	
**Religion n(%)**			
Muslim	769 (85.7%)	128 (14.3%)	0.55
Christian	26 (89.7%)	3 (10.3%)	
**Working beside studying because of income need n(%)**			
Yes	87 (86.1%)	14 (13.9%)	0.93
No	708 (85.8%)	117 (14.2%)	
**Marital Status n(%)**			
Single	751 (85.6%)	126 (14.4%)	0.6
Engaged	40 (88.9%)	5 (11.1%)	
Married	4 (100%)	0 (0%)	

Data presented as no and ℅ calculated by row

^a^Significant

Regarding the factors affecting attitude, the academic year was the only factor that showed a statistically significant difference between appropriate and inappropriate attitudes, *p*=0.05 (Table 3). There was no statistically significant difference between the groups regarding age, sex, university, university categories, academic year categories, academic grade, residency, living conditions, nationality, religion, marital status, and employment status (Table 3).

**Table 3:** Factors affecting attitude towards family planning

	Inappropriate Attitude (*N* = 488)	Appropriate Attitude (*N* = 438)	*p*-value
**Age in Years mean (SD)**	21.36 (1.90)	21.47 (1.89)	0.37
**Gender n(%)**			
Male	242 (54.6%)	201 (45.4%)	0.26
Female	246 (50.9%)	237 (45.4%)	
**Academic year**			
**Basic Years n(%)**	144 (52%)	133 (48%)	0.41
1st year	71 (55.5%)	57 (44.5%)	0.05^a^
2nd year	73 (49%)	76 (51%)	
**Clinical Years n(%)**	316 (53.8%)	271 (46.2%)	
3rd year	106 (61.3%)	67 (38.7%)	
4th year	82 (46.9%)	93 (53.1%)	
5th year	77 (50%)	77 (50%)	
6th year	51 (60%)	34 (40%)	
**Interns n(%)**	28 (45.2%)	34 (54.8%)	
**University**			
**Governmental**	379 (54.6%)	315 (45.4%)	0.07
Alexandria	51 (54.3%)	43 (45.7%)	0.5
Aswan	55 (53.4%)	48 (46.6%)	
El Menofia	41 (53.9%)	35 (46.1%)	
Helwan	49 (60.5%)	32 (39.5%)	
Mansoura	65 (52%)	60 (48%)	
PortSaid	19 (59.4%)	13 (40.6%)	
Suez	42 (48.3%)	45 (51.7%)	
Tanta	57 (59.4%)	39 (40.6%)	
**Private**			
MUST	50 (42.7%)	67 (57.3%)	
**Azhar**	59 (51.3%)	56 (48.7%)	
Azhar-Boys	35 (53.8%)	30 (46.2%)	
Azhar-Girls	24 (48%)	26 (52%)	
**Academic Grade n(%)**			
A, A-	271 (53.8%)	233 (46.2%)	0.85
B, B-	171 (52%)	158 (48%)	
C, C-	39 (48.8%)	41 (51.2%)	
D	7 (53.8%)	6 (46.2%)	
**Residency n(%)**			
Rural	303 (51.4%)	286 (48.6%)	0.31
Urban	185 (54.9%)	152 (45.1%)	
**Living Condition n(%)**			
Alone	35 (48.6%)	37 (51.4%)	0.24
With family	397 (54.3%)	334 (45.7%)	
With friends/dorms	52 (46.4%)	60 (53.6%)	
Other	4 (36.4%)	7 (63.6%)	
**Nationality n(%)**			
Egyptian	465 (53.2%)	409 (46.8%)	0.21
Other	23 (44.2%)	29 (55.8%)	
**Religion n(%)**			
Muslim	471 (52.5%)	426 (47.5%)	0.52
Christian	17 (58.6%)	12 (41.4%)	
**Working beside studying because of income need n(%)**			
Yes	56 (55.4%)	45 (44.6%)	0.56
No	432 (52.4%)	393 (47.6%)	
**Marital Status n(%)**			
Single	464 (52.9%)	413 (47.1%)	0.87
Engaged	22 (48.9%)	23 (51.1%)	
Married	2 (50%)	2 (50%)	

Data presented as no and ℅ calculated by row

^a^Significant

A Pearson correlation showed the correlation between age, knowledge, and attitude. Knowledge scores appeared to have a significant positive weak correlation with attitude scores ( *r*=0.33, *p*= 0 ). However, the correlation between age and knowledge scores, age and attitude scores wasn’t significant (*r*=0.003, *p*=0.94) (*r*= -0,03, *p*=0.93) respectively.

The regression analysis showed that female sex is a significant predictor of good knowledge (COR=1.47, 95% CI=1.01 - 2.14, *p*= 0.05), indicating that females are 47% more likely to have good knowledge compared to males.

## Discussion

Despite the growing emphasis on family planning worldwide, there remains a critical need to improve knowledge and attitudes towards it in specific countries, particularly Egypt. This study evaluated the Egyptian undergraduate medical students' knowledge and attitude regarding family planning. Interestingly, the gender distribution of our sample was nearly equal and most of them were in clinical years, suggesting that they were at a critical point in their training where practical experience could influence their perspectives on important health topics.

Our findings revealed a concerning portion (85 %) of students showing poor knowledge about family planning, suggesting that the current educational effort may not be sufficient. Remarkably, over half of the participants (54.72%) achieved high academic grades (A or A-) yet reported that 67.2% of their family planning knowledge came from their university curriculum. This shows that high academic performance does not necessarily reflect sufficient knowledge, and spots the light on the need for enhancements in how family planning topics are integrated into medical education. Additionally, the reliance on social media as a source of information was alarming, with 32.5% of students citing it as their primary resource. This underscores the need for increased awareness and reliable educational resources and indicates a problem with the medical students’ learning methods about this topic or a lack of clinical practice in primary healthcare facilities, especially in light of the rising population issues in Egypt.

When comparing our results with previous literature, a study conducted at a university in Malaysia reported a 56.1% level of poor knowledge and showed better knowledge levels than ours [30]. Most of their good knowledge percentage was of Chinese students 54.3% followed by Indian 42.1% then Malay 38.5%, which might be linked to the differences in religious values and cultural norms regarding sexuality in Malaysia [30, 31]. However, a study on female final-year undergraduates at the University of Ibadan, Nigeria revealed that only 22% were aware of reproductive life plans, which is significantly lower than the percentage of good knowledge observed in our study [32]. These variations illustrate how cultural and environmental contexts can significantly impact knowledge and attitudes towards family planning across different countries.

Our findings, in agreement with others, indicate that gender significantly affects knowledge, with females achieving higher scores than males [33, 34]. This indicates that women may be more engaged with reproductive health topics due to personal relevance or societal expectations and burdens without enough engagement from their partners [35]. This emphasises the need for targeted educational interventions that encourage all students, especially males, to engage more deeply with family planning topics. However, this finding contrasts with other studies which found no significant gender difference [36, 37]. Previous research identified negative male perceptions of family planning influenced by societal norms, lack of interest, prevailing perceptions around childbearing, religious beliefs, and concerns about health risks associated with contraceptive methods [38, 39]. Additionally, it was noted that family planning promotion often does not explicitly target men, and existing services are perceived as not meeting their needs [40]. So, educational strategies could be tailored to enhance male students’ engagement and understanding of family planning.

Contrarily, studies on Malaysian medical students and South African university students reported a predominance of male knowledge, which may be because of increased targeted educational efforts and services in those areas to address barriers facing males and improve their understanding of family planning [30, 39].

Moreover, we assessed attitude scores and found that 52.7% of Egyptian medical students exhibited mostly inappropriate attitudes toward family planning. Studies conducted on other medical students in Malaysia and India also reported inappropriate attitudes [30, 36, 37, 41], while others in India reported more appropriate ones among interns and post-graduates [42, 43]. The positive attitudes observed in the latter studies can largely be attributed to their focused assessment of a specific aspect of family planning methods, namely emergency contraception. It is a method used to prevent pregnancy after unprotected sex or contraceptive failure, typically involving pills or a copper intrauterine device (IUD) taken within a few days after the event [15]. Their positive attitude is quietly anticipated as unintended pregnancy hugely burdens a female’s social status, in contrast to the positive experience of pregnancy among married females as reported by other authors [44]. While the inappropriate attitudes students have toward family planning and contraception may be because of their poor knowledge, and belief of the harmful effects of contraceptives as being unreliable, causing cancer, decreasing sexual pleasure, and increasing illegal intercourse [45]. Other studies on university students found almost the same prevalence of knowledge and attitude as ours [32, 46, 47], while others disagreed [33, 34].

Surprisingly, many sociodemographic factors, such as age, religion, marital status and university type, did not significantly influence knowledge or attitudes. This suggests that the challenges in understanding and accepting family planning are widespread among students, regardless of their backgrounds.

Regarding attitude in our study, there was a significant difference among participants based on the academic year. This is supported by the results of others who found that the academic year significantly predicts appropriate attitudes [36, 37]. It’s encouraging to see that students in later academic years are showing more appropriate attitudes, likely due to increased exposure to clinical practice. This highlights the importance of engaging with real patients and situations which can shape more positive views.

Our analysis revealed a significant positive weak correlation between knowledge and attitude scores which agrees with other studies [34, 36, 37, 47, 48]. This is a hopeful sign stating that if we can improve knowledge through effective education, we may also improve attitudes. Correlations between age and knowledge scores, age and attitude scores were both insignificant, which is in agreement with another study [36]. This may imply that educational interventions or societal influences could be more critical in affecting these outcomes than the participants’ age alone.

### Strengths and limitations

Our study is a multicentric study and the first to be conducted about knowledge and attitudes toward family planning among medical students in Egypt. It is also one of the pioneers’ studies to use the multistage random methodology to represent this topic. We analyzed medical students’ responses from all college years and compared their different levels of knowledge and attitude. We achieved a large sample size enough for the results to be generalizable to our target population. However, it has some limitations. We have not assessed the families’ socioeconomic status or their practice of family planning methods which may affect students’ knowledge and attitude levels. We also did not have enough representation of Christians to assess the effect of religion, or enough married couples to assess the effect of marital status. The cross-sectional nature of the study restricts drawing definitive conclusions about cause and effect. In addition, relying on self-reported data from questionnaires can introduce biases. So, we suggest conducting more longitudinal research to study this relationship, increase knowledge, and improve the attitudes of medical students and future healthcare providers.

### Conclusion

The overall scores of knowledge and attitude towards family planning were low. Female gender was a significant predictor of good knowledge. Academic year significantly affected the attitude with higher appropriate attitudes found among 4th-year students. Stakeholders concerned with family planning should work together to bring behavioral changes towards family planning by providing information, education and communication.

### Implications for future practice and recommendations

More studies should focus on assessing the levels of knowledge of family planning among medical students, as well as dentistry, pharmacy, and paramedical students. Expanding our research to include a more diverse range of students across different regions and socioeconomic backgrounds would enhance the generalizability of our findings. We should also investigate specific educational interventions to see the best method for improving knowledge and attitudes toward family planning. Future research should consider longitudinal studies to see how knowledge and attitudes change over time, as well as qualitative methods to better understand the reasons behind these trends. In addition, medical educators should assess the resources from which medical students learn about family planning. Accordingly, educational programs about family planning should be integrated early into medical curricula as a crucial component of comprehensive healthcare training. An effective program should not only cover basic knowledge about family planning but also emphasize the responsibilities of healthcare professionals in promoting and implementing family planning practices A recent study in the USA showed that broadening reproductive health education in medical school will prepare future primary care physicians to discuss the full range of reproductive options for their patients [49]. Additionally, active educational techniques, such as clinical scenarios that simulate real-life situations, awareness campaigns, and interactive learning workshops, would enhance teaching effectiveness. These programs should extend into the clinical years of medical education and connect proper family planning awareness with various clinical conditions.

## Supplementary Information

Supplementary Material 1.

## Data Availability

Datasets used during this study are available from the corresponding author upon reasonable request.
