# Supplementary material for: Knowledge and attitude on family planning among medical students in Egypt: a multicentric cross-sectional study
Source: BMC Public Health. 2025 May 28;25:1966. doi: 10.1186/s12889-024-20827-9 (PMC12117728; doi:10.1186/s12889-024-20827-9)
Supplement: Supplementary file 1 — Supplementary Material 1. [file 12889_2024_20827_MOESM1_ESM.docx]

**Section 1: Sociodemographic Characteristics**

**Age** (18:20 – 21:23 – 23:25 – 26:28)

**Gender** (male – female)

**Academic year** (1^st^ year – 2^nd^ year – 3^rd^ year – 4^th^ year – 5^th^ year – 6^th^ year – Intern)

**University** (Mansoura – Tanta – ElMenofia – Aswan - Port Said – Alexandria – Helwan - Suez Canal - Misr University(MUST) - El Azhar Cairo boys - El Azhar Cairo girls)

**Residency** (Urban – Rural)

**Living condition** (Alone – with family – with friends/dorms – other)

**Nationality** (Egyptian – Other)

**Religion** (Muslim – Christian – other)

**Do you work beside studying because of income need?** (Yes – No)

**Marital status** (Single – engaged – married)

**If you are married, how many children (if any) do you have?** (0 – 1- 2- 3 – More than 3)

**Section 2: Knowledge Questions**

**Definition (Yes – No - Don’t know)**

Family planning means spacing the birth of children.

Family planning is the same as abortion.

Family planning kills babies.

Family planning is a decision of both husbands

Natural Family Planning method is a way of preventing pregnancy without the use of drugs or devices.

A condom is a rubber that is inserted into the penis before sexual intercourse.

Irregular menstruation is one of the possible side effects of IUD.

A pill is taken by a woman once a week to prevent pregnancy.

The pills prevent pregnancy by stopping the release of the sperm from the testes.

Depo Provera injection is given to a woman every month to prevent pregnancy.

The effect of injection is still present up to 4 months even though injection has been stopped.

A condom prevents pregnancy by keeping the sperm from getting into the vagina.

An IUD can travel to the different parts of the body.

Injection causes abnormal or deformed babies.

The string of an IUD traps the penis during sexual intercourse Pills cause cancer.

IUD prevents pregnancy by blocking the sperm to come in contact with the egg cell.

Vasectomy decreases a man’s sexual satisfaction.

A woman who has undergone tubal ligation cannot do heavy work.

Tubal ligation involves tying and cutting of both fallopian tubes of a woman.

A woman who is ligated will stop menstruating.

Vasectomy is a simple operation that makes a man sterile.

Vasectomy prevents pregnancy by blocking the sperm from reaching the vagina.

Vasectomy is a simple operation that makes a man sterile.

**Section 3: Attitude Questions**

**Statements (SA – A - NA/ND – D - SD)**

Do you want to know more about family planning?

Are you willing to practice family planning?

Family planning is only for young couples.

Bringing up a family is a shared responsibility of both husband and wife.

Family planning improves maternal and child health.

Family planning is harmful.

Husbands should also participate in family planning decisions.

Family planning is important in ensuring a healthy family.

OCPs can prevent ovarian cysts.

Depo Provera is suitable for couples at beginning marriage Condom can prevent STDs.

Emergency Contraception is just usable for condom failure.

Emergency Contraception is usable for unprotected intercourse.

Weight gain and nausea are OCP’s side effects.

IUD is suitable for forgetful women.

**SA: Strongly Agree A: Agree NA/ND: Not Agree nor Disagree D: Disagree SD: Strongly Disagree**
